# Maternal Bariatric Surgery and Offspring Health: A Sibling Matched Analysis Comparing Offspring Born before and after the Surgery †

**DOI:** 10.3390/jcm12093056

**Published:** 2023-04-23

**Authors:** Itamar Gothelf, Eyal Sheiner, Tamar Wainstock

**Affiliations:** 1 Goldman Medical School, Faculty of Health Sciences, Ben-Gurion University of the Negev, Beer-Sheva 8410501, Israel; gothelf@post.bgu.ac.il; 2 Department of Obstetrics and Gynecology, Soroka University Medical Center, Ben-Gurion University of the Negev, Beer-Sheva 8410501, Israel; sheiner@bgu.ac.il; 3 School of Public Health, Faculty of Health Sciences, Ben-Gurion University of the Negev, Beer-Sheva 8410501, Israel

**Keywords:** bariatric surgery, maternal obesity, long-term follow-up, offspring morbidities, sibling analysis, retrospective cohort

## Abstract

(1) Background: Due to the global escalation in the prevalence of obesity, bariatric surgeries have become a popular solution in many western countries. The aim of the current study was to compare offspring health and the obesity of women before and after undergoing bariatric surgeries. (2) Methods: A retrospective population-based study was performed, including all singleton deliveries which occurred at a tertiary medical center between the years 1991–2021. Among women who had bariatric surgeries, the health of the offspring born before and after the surgery were compared. The offspring were followed up until the age of 18 years, and their hospitalization records were summarized. The incidence of hospitalization with either endocrine, cardiac, respiratory, neurologic, or infectious diagnoses were compared between the groups, as well as offspring obesity. Multivariable Cox proportional models were used to match between siblings and to address confounding variables, including maternal age, gestational age at delivery, fertility treatment, smoking and pregnancy hypertensive disorders. (3) Results: The study population included 3074 deliveries of women who underwent bariatric surgeries (1586 were before and 1488 after the surgery). Offspring born after maternal bariatric surgeries were at a comparable risk for most morbidities, besides endocrine-related morbidities (3.1% vs. 5.0%, OR = 1.61; 1.1–2.35) and obesity (2.5% vs. 4.1%, OR = 1.63; 1.08–2.48). The risk for these morbidities was higher among the offspring of mothers after, vs. before, the surgery, despite adjustment for maternal age and other confounding variables. (4) Conclusions: While bariatric surgeries are considered an effective treatment for obesity, it seems to have less of an effect on the offspring of women who underwent such surgeries. Other persistent factors are most likely associated with the offspring’s risk for morbidities, especially endocrine morbidities and obesity, which remain even though the mother underwent bariatric surgeries.

## 1. Introduction

Over 650 million adults worldwide suffer from obesity, the most prevalent medical condition among women of reproductive age [1]. Maternal obesity, defined as pregnancy body mass index (BMI) ≥ 30 kg/m^2^ increases complications for both mother and offspring during pregnancy and infancy. Maternal risks include a higher likelihood of hypertensive disorders, gestational diabetes, preeclampsia, postpartum hemorrhage, and caesarean delivery [2,3,4,5]. The newborns are at an increased risk of being large for gestational age (LGA), neonatal intensive-care unit admission and perinatal death [6,7,8]. For these reasons, obese women trying to conceive are encouraged to lose weight by modifying their diet and increasing physical activity. When conservative approaches are not sufficient, particularly in cases of severe obesity, surgical options are considered [9,10].

In bariatric surgery, the digestive tract is altered to limit food intake and modify digestion processes so that fewer calories are absorbed [11]. Common bariatric procedures include sleeve gastrectomy (SG), Roux-en-Y gastric bypass (RYGB) and laparoscopic adjustable gastric band (LAGB). RYGB is known for its long-term sustainable weight loss [12] although SG is becoming more popular, and is recommended as the primary procedure in cases of future family planning [13]. However, there is no definite evidence to determine the most appropriate surgical procedure in terms of improved pregnancy-related outcomes [14,15].

Considering the major increase in bariatric surgeries in the last decades [16], and given the negative impact of obesity on pregnancy, it is unsurprising that pre-pregnancy bariatric surgeries are a growing field of interest regarding the possible effects on pregnancy outcomes [17]. Several studies have examined pregnancy outcomes following bariatric surgery and have demonstrated a reduced incidence of gestational diabetes, hypertensive disorders, preeclampsia, and LGA infants [18,19,20,21]. Despite these benefits, bariatric surgery can result in nutritional deficiencies, such as deficiencies in folate, iron, vitamin D, which may have maternal and fetal implications. Studies have reported an increased risk of small-for-gestational age (SGA) neonates, preterm birth, and neonatal intensive-care unit hospitalization among pregnancies following bariatric surgeries. These studies compared pregnant women who underwent bariatric surgery prior to pregnancy with pregnant women who were eligible for bariatric surgery but did not undergo the procedure [22,23].

While maternal bariatric surgery has been extensively researched for its effects on obstetrical and short-term infant outcomes, limited studies have investigated its long-term impact. Some small-scale sibling -nalysis studies, comparing siblings born before and after maternal bariatric surgery, have reported lower rates of obesity in children (BMI at or above the 95th percentile for age and sex) born after maternal surgery [24,25]. Another small-scale sibling analysis study found significant metabolic improvements, including improved insulin sensitivity, lipid profiles, lower c-reactive protein levels, and increased ghrelin levels [26]. However, some studies have failed to demonstrate any effect on weight development and have even suggested an increased risk for long-term pediatric endocrine morbidity [27,28]. These studies typically suffer from small sample sizes, limiting their generalizability. Moreover, most studies focus only on endocrine morbidities, mainly obesity, and do not provide a comprehensive understanding of the overall health outcomes of the offspring.

The aim of this study was to investigate the long-term effects of maternal bariatric surgery on offspring health outcomes. In this study, we utilized an exclusively large-scale sibling-analysis approach in contrast to other studies. This study design allowed for a direct comparison of siblings born before and after maternal bariatric surgery, thus controlling for shared genetic and environmental factors.

## 2. Experimental Section

### 2.1. Study Design

A population-based sibling-analysis retrospective cohort study was performed. The study population included mothers who underwent a bariatric surgery and had at least one delivery before, and at least one delivery after, the surgery. All offspring of the mothers who underwent the surgery were included, and the comparison was performed between offspring born before and after the surgery. All deliveries occurred at Soroka University Medical Center (SUMC), the only tertiary hospital in the southern region of Israel, between 1991 and 2021. Multiple gestations were excluded from the analysis.

The data for this study were collected from two databases: the computerized perinatal database of the Obstetrics and Gynecology department at SUMC, which includes information documented during pregnancy and following delivery, and consists of information recorded immediately following delivery by an obstetrician, and the computerized pediatric database of SUMC, which includes medical diagnosis during offspring’s hospitalizations. The two databases were cross-linked and merged based on mother and infant identification numbers. Skilled medical secretaries regularly review the information before entering it into the database to ensure accuracy and completeness. Maternal and offspring morbidities were defined using a preexisting list of diagnoses by the International Classification of Diseases, ninth/tenth version codes (ICD-9 or 10).

The dependent variable in this study was offspring morbidities up to the age of 18 years old. The morbidities were categorized into the following six groups: endocrine (and specifically offspring Obesity, defined as weight >95% percentile per age and sex); cardiac; respiratory; infectious; neurologic; and malignancies. The list of all diagnoses included, by categories of morbidities, is presented in Supplementary Table S1. Offspring were followed up until they reached 18 years of age, until the end of the study period (2021), or until they were hospitalized for the first time with any of the diagnoses from the mentioned list, separately, for each of the six categories.

The independent variable was defined as the timing of delivery, offspring born before vs. after maternal surgery.

The background characteristics consisted of: (1) maternal characteristics, including age, ethnicity, smoking; (2) obstetric characteristics, including pregnancy-related hypertensive disorder, diabetes, insufficient prenatal care, fertility treatments, intra-uterine growth restriction (defined as a single measurement of estimated fetal weight <10th percentile for gestational age, or head to abdomen circumference ratio > 1), induced labor, placental abruption, placenta previa; and (3) labor and perinatal characteristics, including gestational age at delivery, cesarean delivery, shoulder dystocia, prolonged second stage, gender, birthweight, small for gestational age, large for gestational age, preterm delivery, malformation, 5 min Apgar score < 7, mortality.

### 2.2. Statistical Analysis

A univariate analysis was conducted to compare perinatal and delivery characteristics of newborns born before and after maternal bariatric surgery. In this section the results are not matched by siblings. Categorical variables, such as pregnancy-related hypertensive disorder, gestational diabetes mellitus, fertility treatments, and cesarean delivery were compared using the chi-squared test, while continuous variables, such as birthweight, maternal age, and gestational age at delivery, were compared using the *t*-test. Cumulative incidence rates of offspring hospitalizations before and after maternal bariatric surgery were compared using Kaplan–Meier and log–rank tests to determine significant differences.

A multivariable Cox proportional regression model was performed to study the association between maternal bariatric surgeries (as compared to their siblings, born before the surgeries) and offspring health. The models were used to calculate the Hazard ratios and their 95% confidence intervals (CI) and accounted for the siblings and maternal recurrence in the cohort, while adjusting for confounders that were found to be significantly different between the two study groups (before and after the surgery) and clinically significant variables, including maternal age, gestational age at delivery, fertility treatment, smoking, and pregnancy-related hypertensive disorders. Statistical significance was set at *p* < 0.05. The statistical analysis was performed using SPSS software (version 29th).

## 3. Results

A total of 395,408 deliveries occurred (by 135,332 women) at Soroka Medical Center between 1991–2021. After excluding 15,150 multiple gestations, 380,258 deliveries remained. There were 1086 women who underwent bariatric surgeries during the study period. These women had 1586 deliveries before their surgeries, and 1488 deliveries after them. The study population therefore included 3074 offspring. Table 1 provides detailed information on pregnancy characteristics and delivery outcomes across the study groups. Mothers were older after the bariatric surgery (31.3 ± 5.2 vs. 27.3 ± 5.5) and were more likely to smoke (4.0% vs. 2.6%). Following the surgery, there was a significant decrease in the risk for pregnancy-related hypertensive disorders (10.5% vs. 15.4%). There was no significant change in incidence of gestational diabetes mellitus following the surgery. After the surgery, mothers were more likely to have a cesarean delivery (37.4% vs. 24.7%) at an earlier gestational age (38.5 ± 2.1 vs. 38.8 ± 2.5 gestational weeks), although rates of preterm deliveries were not significantly higher following the bariatric surgery. Rates of large-for-gestational-age were lower following the surgery (4.8% vs. 8.4%), as was the risk for perinatal mortality (0.9% vs. 2.3%).

Table 2 summarizes the differences in long-term morbidities between offspring of mothers before and after bariatric surgery. No differences were found between the groups in hospitalization with any of the following morbidities: cardiac, respiratory, infectious, neurologic, or malignancies. However, offspring born to mothers following the surgery had significantly higher rates of endocrine morbidity hospitalizations (5.0% vs. 3.1%, OR = 1.61 *p* = 0.02), and specifically, they were more likely to be obese (4.1% vs. 2.5%, OR = 1.63 *p* = 0.02).

**Figure 1:**
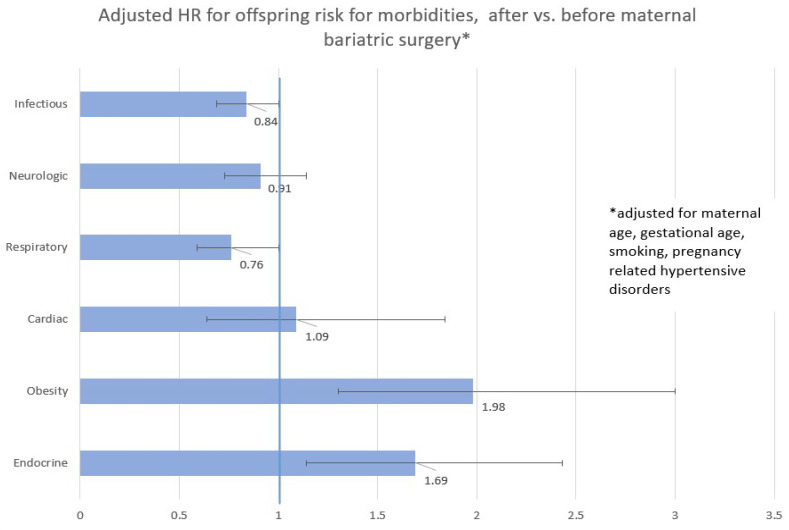
Results of multivariable Cox proportional adjusted models regarding offspring long-term morbidities after vs. before maternal bariatric surgery.

Figure 1 presents the Hazard ratios and 95%CI for the association between being born following bariatric surgeries (vs. before) and results of the multivariable Cox proportional models. All models were adjusted for maternal age, gestational age, smoking, and pregnancy-related hypertensive disorders. Offspring born to women following bariatric surgery were at an increased risk for endocrine-related hospitalization (adjusted HR = 1.69, 95%CI 1.14–2.43), and were more likely to be obese (adjusted HR = 1.98, 95%CI 1.30–3.00).

## 4. Discussion

In this large population based retrospective cohort study, which applied a sibling matched analysis, offspring born to mothers who underwent bariatric surgery were not found to be at a lower risk for morbidities as compared to siblings born before the mother underwent the surgery; in fact, they were at an even increased risk for endocrine complications throughout childhood.

Previous studies have shown that bariatric surgery can reduce the risk of obstetrical complications such as gestational diabetes, pregnancy-related hypertension, macrosomia, and preeclampsia [29]. Consequently, it would be expected that bariatric surgery would also reduce the long-term risk of morbidities in offspring. However, the results of this study are in contrast to these expectations, which were consistent with two prior studies that reported an increased risk of obesity and endocrine complications in children born to mothers who underwent bariatric surgery. Willmer et al. and Damti et al. found no reduced risk for offspring obesity in newborns born after the surgery, or even an increased risk for long-term pediatric endocrine morbidity [27,28]. Barisione et al., however, found a slight decrease in obesity rates of offspring born after maternal bariatric surgery compared to sibling offspring born before maternal surgery. Data in the latter study were based on self-reporting (telephone survey), and in some cases after a long period, and therefore require careful consideration [25].

A possible explanation for the increased risk of obesity and endocrine morbidity may be the lack of breastfeeding, which is known to have numerous health benefits, including a reduced risk of childhood obesity [30,31]. Exclusive breastfeeding rates appear to be minimal in the group of post-surgery women, with one study revealing that 96% percent of women after bariatric surgery were no longer exclusively breastfeeding within five days of giving birth [32]. Maternal concerns regarding the nutritional adequacy of breastmilk following bariatric surgery may be associated with the low breastfeeding rates among these women. These women may also cease breastfeeding due to excess skin from significant weight loss, which can change breast shape and may impact the infant’s ability to successfully breastfeed [33]. Therefore, newborns born after maternal surgery are less likely to be breastfed, increasing their risk for obesity.

Bariatric surgery has been linked to alterations in epigenetic markers in the mother’s genome, and also affects genes expression in the offspring [34]. Epigenetic changes may occur in the offspring due to exposure to an adverse in utero environment, which may be induced following the bariatric surgery due to nutritional deficiencies, resulting in the altered structure and function of different organs and control systems [35]. Maternal epigenetic predisposition may also lead to inter-generational passage and affect the expression of genes in the offspring [28]. Liu et al. found that maternal obesity was associated with offspring DNA methylations in genes involved with inflammation-mediated disorders [36]. Consequently, these changes may have a long-lasting impact on offspring’s metabolic health.

Another possible explanation for the observed results may be the lower birth weight and higher proportion of small-for-gestational-age (SGA) among newborns after, vs. before, maternal surgery [37]. SGA newborns are known to experience rapid weight gain during infancy (catch-up growth), and both are associated with an increased risk of obesity in later life [38]. In order to prevent nutritional deficiencies in post-surgery patients, patients are typically advised to increase their caloric intake. Infants born after maternal bariatric surgery may be fed more, gain more weight and therefore increase their risk for obesity later in life compared to their counterparts born before maternal surgery [25].

Maternal obesity has been associated with an elevated risk of respiratory morbidity in early childhood, including asthma, respiratory infection and bronchiectasis, as demonstrated in prior studies, [39,40,41] as well as cardiovascular morbidities, such as hyperlipidemia, elevated blood pressure, insulin resistance, and elevated inflammatory markers [42,43]. Therefore, bariatric surgery in obese mothers was expected to lower the likelihood of such morbidities in their children. However, our study found no association between maternal bariatric surgery and respiratory or cardiovascular morbidities in offspring, and further studies are recommended to clarify this matter.

The main strength of this study is the large-scale sibling analysis, comparing siblings born before and after maternal surgery, used to examine long-term morbidities while minimizing the influence of genetic, cultural, and behavioral factors. Furthermore, to the best of our knowledge, this is the first study to examine the effects of maternal bariatric surgery on a range of offspring long-term morbidities throughout childhood. An additional strength of the study is that it was based on the Soroka University Medical Center database, which serves the entire population of southern Israel, offering both maternity and pediatric services, thus ensuring a comprehensive assessment of offspring morbidity.

Several study limitations need to be addressed. One is the lack of data on the fathers in the cohort, and the possibility of different fathers between the siblings. This may have affected the genetic factors, which we assumed were similar in siblings. Additionally, data regarding the types of bariatric procedures were unavailable. The type of surgical procedure performed may have affected maternal weight loss, potentially affecting offspring long-term morbidity. Furthermore, data on important factors such as BMI, maternal weight reduction following bariatric surgery, and time since the procedure, were not available and were not addressed, which could have provided additional insights into the results. Breastfeeding is another important factor that was not addressed in our study and may have an impact on offspring morbidities, and specifically endocrine morbidities and obesity, as previously mentioned.

## 5. Conclusions

This large, population-based sibling analysis contributes to our understanding of the benefits and risks of maternal bariatric surgery. Additional research is recommended to fully comprehend the extended effect of maternal bariatric surgery on offspring long-term health, and to identify feasible strategies to reduce these risks. It is important for healthcare providers to be aware of these outcomes and provide suitable counseling and support to women who are expecting, or planning to conceive, after undergoing bariatric surgery.

## Figures and Tables

**Table 1:** Characteristics and delivery outcomes of the study population, after vs. before maternal bariatric surgery *.

	Before Bariatric Surgery *n* = 1586 (51.6%)	After Bariatric Surgery *n* = 1488 (48.4%)	Odds Ratio; 95%CI, *p*
Maternal age (mean ± SD)	27.3 ± 5.5	31.3 ± 5.2	*p* < 0.001
Ethnicity			
Bedouin	466 (29.4)	217 (14.6)	2.44; 2.04–2.92; *p* < 0.001
Jewish	1120 (70.6)	1271 (85.4)	
Pregnancy related hypertensive disorders	245 (15.4)	156 (10.5)	0.64; 0.52–0.79; *p* < 0.001
Gestational Diabetes mellitus	173 (10.9)	161 (10.8)	0.99; 0.79–1.24; *p* = 0.95
Smoking	38 (2.4)	59 (4.0)	1.68; 1.11–2.54; *p* = 0.01
Insufficient of prenatal care	55 (3.5)	36 (2.4)	0.69; 0.45–1.06; *p* = 0.09
Fertility treatments	72 (4.5)	89 (6.0)	1.34; 0.97–1.84; *p* = 0.08
Intra-uterine growth restriction	31 (2.0)	46 (3.1)	1.6; 1.0–2.54; *p* = 0.05
Induced labor	599 (37.8)	459 (30.8)	0.74; 0.63–0.85; *p* < 0.001
Placental abruption	6 (0.4)	8 (0.5)	1.42; 0.49–4.11; *p* = 0.6
Placenta previa	5 (0.3)	7 (0.5)	1.5; 0.47–4.72; *p* = 0.57
Malformation	93 (5.9)	120 (8.1)	1.41; 1.06–1.86; *p* = 0.02
Gestational age at delivery (mean ± SD)	38.8 ± 2.5	38.5 ± 2.1	*p* = 0.004
Cesarean delivery	391 (24.7)	557 (37.4)	1.83; 1.57–2.14; *p* < 0.001
Post partum hemorrhage	10 (0.6)	7 (0.5)	0.75; 0.28–1.96; *p* = 0.63
Gender of offspring			
Male	811 (51.1)	772 (51.9)	1.03; 0.89–1.19; *p* = 0.69
Female	775 (48.9)	716 (48.1)	
Birthweight (kg, mean ± SD)	3.248 ± 0.62	3.128 ± 0.551	*p* < 0.001
Low birthweight (<2.500 kg)	137 (8.6)	147 (9.9)	1.16; 0.91–1.48; *p* = 0.24
Small for gestational age	59 (3.7)	71 (4.8)	1.30; 0.91–1.85; *p* = 0.15
Large for gestational age	134 (8.4)	72 (4.8)	0.55; 0.41–0.74; *p* < 0.001
Preterm delivery (<37 gestational weeks)	136 (8.6)	146 (9.8)	1.16; 0.91–1.48; *p* = 0.24
Preterm delivery (<34 gestational weeks)	49 (3.1)	32 (2.2)	0.69; 0.44–1.08; *p* = 0.12
5 min Apgar score < 7	16 (1.0)	22 (1.5)	1.46; 0.76–2.79; *p* = 0.26
Mortality	36 (2.3)	13 (0.9)	0.38; 0.2–0.72; *p* = 0.002

* All numbers represent the *n* and (%), unless otherwise stated. Rates were compared using chi-square test, and the means compared using *t*-tests.

**Table 2:** Differences in long-term morbidities between offspring of mothers before and after bariatric surgery.

Offspring Morbidities up to the Age of 18	Before Bariatric Surgery *n* = 1493 (52.2%)	After Bariatric Surgery *n* = 1368 (47.8%)	Odds Ratio; 95%CI
Endocrine	47 (3.1)	68 (5.0)	1.61; 1.1–2.35; *p* = 0.02
Obesity	38 (2.5)	56 (4.1)	1.63; 1.08–2.48; *p* = 0.02
Cardiac	24 (1.6)	26 (1.9)	1.19; 0.68–2.08; *p* = 0.57
Respiratory	181 (12.1)	158 (11.5)	0.95; 0.75–1.19; *p* = 0.64
Neurologic	178 (11.9)	174 (12.7)	1.08; 0.86–1.35; *p* = 0.53
Infectious	524 (35.1)	472 (34.5)	0.97; 0.84–1.14; *p* = 0.75
Malignancy	7 (0.5)	13 (1.0)	2.04; 0.81–5.12; *p* = 0.18

## Data Availability

Data will be made available by request, and pending on IRB approval.

## Supplementary Materials

The following supporting information can be downloaded at: https://www.mdpi.com/article/10.3390/jcm12093056/s1, Table S1: Diagnoses by categories of morbidities.
