# Supplementary material for: Maternal Bariatric Surgery and Offspring Health: A Sibling Matched Analysis Comparing Offspring Born before and after the Surgery
Source: J Clin Med. 2023 Apr 23;12(9):3056. doi: 10.3390/jcm12093056 (PMC10179329; doi:10.3390/jcm12093056)
Supplement: Supplementary file 1 [file jcm-12-03056-s001.zip › jcm-2350048-supplementary.pdf]

Diagnosis (	Diagnosis Description
**Cardiac diagnoses**	
3940	MITRAL STENOSIS
3949	OTHER AND UNSPECIFIED MITRAL VALVE DISEASES
3961	MITRAL VALVE STENOSIS AND AORTIC VALVE INSUFFICIENCY
3963	MITRAL VALVE INSUFFICIENCY AND AORTIC VALVE INSUFFICIENCY
3968	MULTIPLE INVOLVEMENT OF MITRAL AND AORTIC VALVES
3970	DISEASES OF TRICUSPID VALVE
4240	MITRAL VALVE DISORDERS
4241	AORTIC VALVE DISORDERS
4242	TRICUSPID VALVE DISORDERS, SPECIFIED AS NONRHEUMATIC
4243	PULMONARY VALVE DISORDERS
4019	UNSPECIFIED ESSENTIAL HYPERTENSION
40390	UNSP. HYPERTENSIVE KIDNEY DIS. WITH CHRONIC KIDNEY DISEASE STAGE I THROUGH STAGE I
40391	UNSP. HYPERTENSIVE KIDNEY DIS. WITH CHRONIC KIDNEY DISEASE
40391	UNSP. HYPERTENSIVE KIDNEY DIS. WITH CHRONIC KIDNEY DISEASE STAGE V OR END STAGE RE
40391	UNSP. HYPERTENSIVE RENAL DIS.+ RENAL FAILURE
40591	UNSPECIFIED RENOVASCULAR HYPERTENSION
4260	ATRIOVENTRICULAR BLOCK, COMPLETE
42611	FIRST DEGREE ATRIOVENTRICULAR BLOCK
42612	MOBITZ (TYPE) II ATRIOVENTRICULAR BLOCK
42613	OTHER SECOND DEGREE ATRIOVENTRICULAR BLOCK
4263	OTHER LEFT BUNDLE BRANCH BLOCK
4264	RIGHT BUNDLE BRANCH BLOCK
4267	ANOMALOUS ATRIOVENTRICULAR EXCITATION
4267 1	WOLFF-PARKINSON-WHITE SYNDROME
42682	LONG QT SYNDROME
42689	OTHER SPECIFIED CONDUCTION DISORDERS
4270	PAROXYSMAL SUPRAVENTRICULAR TACHYCARDIA
4271	PAROXYSMAL VENTRICULAR TACHYCARDIA
4272	PAROXYSMAL TACHYCARDIA, UNSPECIFIED
4273	ATRIAL FIBRILLATION AND FLUTTER
42731	ATRIAL FIBRILLATION
42732	ATRIAL FLUTTER
42741	VENTRICULAR FIBRILLATION
4275	CARDIAC ARREST
42760	PREMATURE BEATS, UNSPECIFIED
42761	SUPRAVENTRICULAR PREMATURE BEATS
42769	OTHER PREMATURE BEATS
427811	SINUS BRADYCARDIA
42789	OTHER SPECIFIED CARDIAC DYSRHYTHMIAS
4279	CARDIAC DYSRHYTHMIA, UNSPECIFIED
7850	TACHYCARDIA, UNSPECIFIED
7851	PALPITATIONS
390	RHEUMATIC FEVER WITHOUT MENTION OF HEART INVOLVEMENT
3911	ACUTE RHEUMATIC ENDOCARDITIS
3918	OTHER ACUTE RHEUMATIC HEART DISEASE
3919	ACUTE RHEUMATIC HEART DISEASE, UNSPECIFIED
3920	RHEUMATIC CHOREA WITH HEART INVOLVEMENT
3929	RHEUMATIC CHOREA WITHOUT MENTION OF HEART INVOLVEMENT

3941	RHEUMATIC MITRAL INSUFFICIENCY
3951	RHEUMATIC AORTIC INSUFFICIENCY
39890	RHEUMATIC HEART DISEASE, UNSPECIFIED
4100	ACUTE MYOCARDIAL INFARCTION OF ANTEROLATERAL WALL
41000	AC. M.I. ANTEROLATERAL, EPISODE OF CARE UNSP.
41011	AC. M.I. OTHER ANTERIOR, INITIAL EPISODE OF CARE
41071	AC. M.I. SUBENDOCARDIAL, INITIAL EPISODE OF CARE
4109	ACUTE MYOCARDIAL INFARCTION OF UNSPECIFIED SITE
41091	AC. M.I. UNSP. SITE, INITIAL EPISODE OF CARE
4111	INTERMEDIATE CORONARY SYNDROME
414	OTHER FORMS OF CHRONIC ISCHEMIC HEART DISEASE
41410	ANEURYSM OF HEART (WALL)
4149	CHRONIC ISCHEMIC HEART DISEASE, UNSPECIFIED
4292	CARDIOVASCULAR DISEASE, UNSPECIFIED
4295	RUPTURE OF CHORDAE TENDINEAE
42979	OTHER, MURAL THROMBUS (ATR.)(VENT.)ACQUIRED,FOLLOWING M.I.
41512	SEPTIC PULMONARY EMBOLISM
41519	OTHER PULMONARY EMBOLISM & INFARCTION
4160	PRIMARY PULMONARY HYPERTENSION
4168	OTHER CHRONIC PULMONARY HEART DISEASES
4169	CHRONIC PULMONARY HEART DISEASE, UNSPECIFIED
4171	ANEURYSM OF PULMONARY ARTERY
42090	ACUTE PERICARDITIS, UNSPECIFIED
42099	OTHER ACUTE PERICARDITIS
4210	ACUTE AND SUBACUTE BACTERIAL ENDOCARDITIS
4211	ACUTE + SUBACUTE INFEC.ENDOCARDITIS IN DIS.CLASS.ELSEWHERE
42290	ACUTE MYOCARDITIS, UNSPECIFIED
42291	IDIOPATHIC MYOCARDITIS
42292	SEPTIC MYOCARDITIS
4230	HEMOPERICARDIUM
4232	CONSTRICTIVE PERICARDITIS
4233	CARDIAC TAMPONADE
4238	OTHER SPECIFIED DISEASES OF PERICARDIUM
4239	UNSPECIFIED DISEASE OF PERICARDIUM
42490	ENDOCARDITIS, VALVE UNSPECIFIED, UNSPECIFIED CAUSE
4251	HYPERTROPHIC OBSTRUCTIVE CARDIOMYOPATHY
4252	OBSCURE CARDIOMYOPATHY OF AFRICA
4253	ENDOCARDIAL FIBROELASTOSIS
4254	OTHER PRIMARY CARDIOMYOPATHIES
4257	NUTRITIONAL AND METABOLIC CARDIOMYOPATHY
4259	SECONDARY CARDIOMYOPATHY, UNSPECIFIED
4289	HEART FAILURE, UNSPECIFIED
4290	MYOCARDITIS, UNSPECIFIED
4280	CONGESTIVE HEART FAILURE
4280	CONGESTIVE HEART FAILURE, UNSPECIFIED
4281	LEFT HEART FAILURE
42841	ACUTE COMBINED SYSTOLIC AND DIASTOLIC HEART FAILURE
42989	OTHER ILL-DEFINED HEART DISEASES
4299	HEART DISEASE, UNSPECIFIED
7852	FUNCTIONAL AND UNDIAGNOSED CARDIAC MURMURS

7852	UNDIAGNOSED CARDIAC MURMURS,(HEART MURMUR NOS)
7852 1	SYSTOLIC MURMUR
7859	OTHER SYMPTOMS INVOLVING CARDIOVASCULAR SYSTEM
**Respiratory diagnoses**	
485	BRONCHOPNEUMONIA, ORGANISM UNSPECIFIED
486	PNEUMONIA, ORGANISM UNSPECIFIED
49300	EXTRINSIC ASTHMA, UNSPECIFIED
49320	CHR. OBSTRUCTIVE ASTHMA, UNSPECIFIED
49321	CHR. OSBTRUCTIVE ASTHMA WITH STATUS ASTHMATICUS
4939	ASTHMA, UNSPECIFIED
49390	ASTHMA,UNSPECIFIED
49390	ASTHMA,UNSPECIFIED TYPE,WITHOUT MENTION OF STATUS ASTHMATICUS
49390	ASTHMA,UNSPECIFIED TYPE,WITHOUT MENTION OF STATUS ASTHMATICUS OR ACUTE EXACEI
49391	ASTHMA, UNSPECIFIED TYPE, WITH STATUS ASTHMATICUS
49392	UNSPECIFIED ASTHMA WITH (ACUTE) EXACERBATION
49392	UNSPECIFIED ASTHMA WITH ACUTE EXACERBATION
496	CHRONIC AIRWAY OBSTRUCTION, NOT ELSEWHERE CLASSIFIED
5060	BRONCHITIS AND PNEUMONITIS DUE TO FUMES AND VAPORS
5100	EMPYEMA WITH FISTULA
5109	EMPYEMA WITHOUT MENTION OF FISTULA
5181	INTERSTITIAL EMPHYSEMA
493	ASTHMA
494	BRONCHIECTASIS
4940	BRONCHIECTESIS WITHOUT ACUTE EXACERBATION
4941	BRONCHIECTASIS WITH ACUTE EXACERBATION
4959	UNSPECIFIED ALLERGIC ALVEOLITIS AND PNEUMONITIS
515	POSTINFLAMMATORY PULMONARY FIBROSIS
5161	IDIOPATHIC PULMONARY HEMOSIDEROSIS
5070	PNEUMONITIS DUE TO INHALATION (FOOD,VOMITUS,OR N.O.S.)
5070	PNEUMONITIS DUE TO INHALATION OF FOOD OR VOMITUS
5071	PNEUMONITIS DUE TO INHALATION OF OILS AND ESSENCES
5078	PNEUMONITIS DUE TO OTHER SOLIDS AND LIQUIDS
5168	OTHER SPECIFIED ALVEOLAR AND PARIETOALVEOLAR PNEUMONOPATHIES
5110	PLEURISY WITHOUT MENTION OF EFFUSION OR CURRENT TUBERCULOSIS
5118	OTHER SPECIFIED FORMS OF PLEURAL EFFUSION, EXCEPT TUBERCULOUS
51181	MALIGNANT PLEURAL EFFUSION
51189	OTHER SPECIFIED FORMS OF EFFUSION, EXCEPT TUBERCULOUS
5119	UNSPECIFIED PLEURAL EFFUSION
5120	SPONTANEOUS TENSION PNEUMOTHORAX
5128	OTHER SPONTANEOUS PNEUMOTHORAX
4928	OTHER EMPHYSEMA
786	SYMPTOMS INVOLVING RESPIRATORY SYSTEM & OTHER CHEST SYMPTOMS
78607	WHEEZING
78609	OTHER DYSPNEA & RESPIRATORY ABNORMALITY
32723	OBSTRUCTIVE SLEEP APNEA (ADULT)(PEDIATRIC)
32727	CENTRAL SLEEP APNEA IN CONDITIONS CLASSIFIED ELSEWHERE
78051	INSOMNIA WITH SLEEP APNEA
78057	OTHER AND UNSPECIFIED SLEEP APNEA
78057	UNSPECIFIED SLEEP APNEA
78051	INSOMNIA WITH SLEEP APNEA, UNSPECIFIED

78609	OTHER DYSPNEA AND RESPIRATORY ABNORMALITY
**Endocrine diagnoses**	
24200	TOXIC DIFFUSE GOITER WITHOUT MENTION OF THYROTOXIC CRISIS
24240	THYROTOXICOSIS FROM ECTOPIC THYROID NODULE,WITHOUT CRISIS
2429	THYROTOXICOSIS WITHOUT MENTION OF GOITER OR OTHER CAUSE
24290	THYROTOXICOSIS WITHOUT GOITER; HYPERTHYROIDISM
243	CONGENITAL HYPOTHYROIDISM
2440	POSTSURGICAL HYPOTHYROIDISM
2443	OTHER IATROGENIC HYPOTHYROIDISM
2448	OTHER SPECIFIED ACQUIRED HYPOTHYROIDISM
2449	UNSPECIFIED ACQUIRED HYPOTHYROIDISM
2450	ACUTE THYROIDITIS
2451	SUBACUTE THYROIDITIS
2452	CHRONIC LYMPHOCYTIC THYROIDITIS
2461	DYSHORMONOGENIC GOITER
2468	OTHER SPECIFIED DISORDERS OF THYROID
24900	SECONDARY DIABETES MELLITUS WITHOUT MENTION OF COMPLICATION, NOT STATED AS UN
24901	SECONDARY DIABETES MELLITUS WITHOUT MENT. OF COMPLI. UNCONTROLLED
2500	DIABETES MELLITUS WITHOUT MENTION OF COMPLICATION
25000	TYPE II/UNSPECIFIED TYPE, DIABETES MELLITUS WITHOUT COMPLICATION, NOT STATED AS UI
25001	JUVENILE TYPE DIABETES MELLITUS WITHOUT MENTION OF COMPLICATION
25001	TYPE I(JUVENILE TYPE), DIABETES MELLITUS WITHOUT COMPLICATION,NOT STATED AS UNCOI
25002	TYPE II OR UNSPECIFIED TYPE, DIABETES MELLITUS, UNCONTROLLED
25003	DIABETES MELLITUS WITHOUT COMPLICATION, TYPE I, UNCONTROLLED
25010	TYPE II OR UNSPECIFIED TYPE, DIABETES MELLITUS WITH KETOACIDOSIS, NOT STATED AS UNC
25011	JUVENILE TYPE DIABETES MELLITUS WITH KETOACIDOSIS
25011	TYPE I (JUVENILE TYPE), DIABETES MELLITUS WITH KETOACIDOSIS, NOT STATED AS UNCONTRO
25013	DIABETES WITH KETOACIDOSIS, TYPE I, UNCONTROLLED
25031	JUVENILE DIABETES, NOT STATED AS UNCONTROLLED, WITH OTHER COMA
25041	TYPE I (JUVENILE TYPE) DIABETES WITH RENAL MANIFESTATIONS NOT STATED AS UNCONTROI
25050	ADULT-ONSET DIABETES,NOT STATED AS UNCONTROLLED,+OPHTALMIC MANIFE
25050	TYPE II OR UNSPECIFIED TYPE,DIABETES WITH OPHTALMIC MANIFESTATION NOT STATED AS U
2508	DIABETES WITH OTHER SPECIFIED MANIFESTATIONS
25080	TYPE II OR UNSPECIFIED TYPE,DIABETES WITH SPECIFIED MANIFESTATION NOT STATED AS UN
25081	JUVENILE DIABETES, NOT STATED AS UNCONTROLLED,+SPEC. MANIFESTATIN
25081	TYPE I(JUVENILE TYPE),DIABETES WITH SPECIFIED MANIFESTATION NOT STATED AS UNCONTR
25083	JUVENILE DIABETES, UNCONTROLLED, + SPEC. MANIFESTATIONS
2510	HYPOGLYCEMIC COMA (NON-DIABETIC INSULIN COMA)
2511	OTHER SPECIFIED HYPOGLYCEMIA
2512	HYPOGLYCEMIA, UNSPECIFIED
64803	ANTEPARTUM DIABETES MELLITUS
2779	UNSPECIFIED DISORDER OF METABOLISM
2780	OVERWEIGHT AND OBESITY
27800	OBESITY, UNSPECIFIED
27801	MORBID OBESITY
27802	OVERWEIGHT
2781	LOCALIZED ADIPOSITY
2519	UNSPECIFIED DISORDER OF PANCREATIC INTERNAL SECRETION
252	DISORDERS OF PARATHYROID GLAND
2520	HYPERPARATHYROIDISM

25200	HYPERPARATHYROIDISM, UNSPECIFIED
25202	SECONDARY HYPERPARATHYROIDISM, NON-RENAL
2521	HYPOPARATHYROIDISM
2532	PANHYPOPITUITARISM
2533	PITUITARY DWARFISM
2535	DIABETES INSIPIDUS
2536	OTHER DISORDERS OF NEUROHYPOPHYSIS
2540	PERSISTENT HYPERPLASIA OF THYMUS
2548	OTHER SPECIFIED DISEASES OF THYMUS GLAND
2550	CUSHING'S SYNDROME
2551	HYPERALDOSTERONISM
25510	HYPERALDOSTERONISM, UNSPECIFIED
25513	BARTTER'S SYNDROME
2552	ADRENOGENITAL DISORDERS
2553	OTHER CORTICOADRENAL OVERACTIVITY
2554	CORTICOADRENAL INSUFFICIENCY
25541	GLUCOCORTICOID DEFICIENCY
25542	MINERALOCORTICOID DEFICIENCY
2555	OTHER ADRENAL HYPOFUNCTION
2558	OTHER SPECIFIED DISORDERS OF ADRENAL GLANDS
2559	UNSPECIFIED DISORDER OF ADRENAL GLANDS
2561	OTHER OVARIAN HYPERFUNCTION
2562	POSTABLATIVE OVARIAN FAILURE
25639	OTHER OVARIAN FAILURE
2564	POLYCYSTIC OVARIES
2572	OTHER TESTICULAR HYPOFUNCTION
2590	DELAY IN SEXUAL DEVELOPMENT/PUBERTY,NOT ELSEWHERE CLASSIFIED
2591	PRECOCIOUS SEXUAL DEVELOPMENT AND PUBERTY, NOT ELSEW.CLASSIF.
25951	ANDROGEN INSENSITIVITY SYNDROME
**Neurological diagnoses**	
2990	AUTISTIC DISORDER
2990	INFANTILE AUTISM
29900	AUTISTIC DISORDER, CURRENT OR ACTIVE STATE
29901	AUTISTIC DISORDER, RESIDUAL STATE
29910	CHILDHOOD DISINTEGRATIVE DISORDER, CURRENT OR ACTIVE STATE
2998	OTHER SPECIFIED PERVASIVE DEVELOPMENTAL DISORDERS
29981	OTHER SPECIFIED PERVASIVE DEVELOPMENTAL DISORDERS, RESIDULA STATE
29990	UNSPECIF.PERVASIVE DEVELOPMENTAL DISORDER,CURRENT OR ACTIVE STATE
3071	ANOREXIA NERVOSA
3075	OTHER AND UNSPECIFIED DISORDERS OF EATING
30750	EATING DISORDER, UNSPECIFIED
30751	BULIMIA NERVOSA
30753	RUMINATION DISORDER
30759	OTHER DISORDERS OF EATING
3073	STEREOTYPIC MOVEMENT DISORDER
30746	SLEEP AROUSAL DISORDER
30746	SOMNAMBULISM OR NIGHT TERRORS
30747	OTHER DYSFUNCTIONS OF SLEEP STAGES OR AROUSAL FROM SLEEP
32730	CIRCADIAN RHYTHM SLEEP DISORDER, UNSPECIFIED
32732	CIRCADIAN RHYTHM SLEEP DISORDER, ADVANCED SLEEP PHASE TYPE

34700	NARCOLEPSY WITHOUT CATAPLEXY
34701	NARCOLEPSY WITH CATAPLEXY
7805	SLEEP DISTURBANCES
78050	UNSPECIFIED SLEEP DISTURBANCE
78052	INSOMNIA, UNSPECIFIED
78052	OTHER INSOMNIA
78054	HYPERSOMNIA, UNSPECIFIED
78056	DYSFUNCTIONS ASSOCIATED WITH SLEEP STAGES OR AROUSAL FROM SLEEP
78057	OTHER & UNSPECIFIED SLEEP APNEA
78059	OTHER SLEEP DISTURBANCES
3331	ESSENTIAL AND OTHER SPECIFIED FORMS OF TREMOR
3332	MYOCLONUS
3335	OTHER CHOREAS
3336	GENETIC TORSION DYSTONIA
3336	IDIOPATHIC TORSION DYSTONIA
33390	UNSP.EXTRAPYRAMIDAL DISEASE + ABNORMAL MOVEMENT DISORDER
33399	OTHER EXTRAPYRAMIDAL DISEASES AND ABNORMAL MOVEMENT DISORDERS
3343	OTHER CEREBELLAR ATAXIA
3450	GENERALIZED NONCONVULSIVE EPILEPSY
34500	GENERALIZED NONCONVULSIVE EPILEPSY WITHOUT INTRACTABLE EPILEPSY
34501	GENERALIZED NONCONVULSIVE EPILEPSY WITH INTRACTABLE EPILEPSY
34510	GENERALIZED CONVULSIVE EPILEPSY WITHOUT INTRACTABLE EPILEPSY
34511	GENERALIZED CONVULSIVE EPILEPSY WITH INTRACTABLE EPILEPSY
3452	PETIT MAL STATUS, EPILEPTIC
3453	GRAND MAL STATUS, EPILEPTIC
34540	PARTIAL EPILEPSY+IMPAIRMENT OF CONSCIOUSNESS WITHOUT INTRACTABLE EPILEPSY
3455	PARTIAL EPILEPSY, WITHOUT IMPAIRMENT OF CONSCIOUSNESS
34550	PARTIAL EPILEPSY WITHOUT IMPAIRMENT OF CONSCIOUSNESS WITHOUT INTR ACTABEL EPILE
3456	INFANTILE SPASMS
34560	INFANTILE SPASMS WITHOUT INTRACTABLE EPILEPSY
3459	EPILEPSY, UNSPECIFIED
34590	EPILEPSY, NUSP. WITHOUT INTRACTABEL EPILEPSY
34590	EPILEPSY, UNSP. WITHOUT INTRACTABLE EPILEPSY
34591	EPILEPSY UNSP. WITH INTRACTABLE EPILEPSY
78031	FEBRILE CONVULSIONS
78031	FEBRILE CONVULSIONS (SIMPLE), UNSPECIFIED
78032	COMPLEX FEBRILE CONVULSIONS
78039	OTHER CONVULSIONS
78099	OTHER GENERAL SYMPTOMS
7810	ABNORMAL INVOLUNTARY MOVEMENTS
7812	ABNORMALITY OF GAIT
7813	LACK OF COORDINATION
3341	HEREDITARY SPASTIC PARAPLEGIA
3421	SPASTIC HEMIPLEGIA
34210	SPASTIC HEMIPLEGIA AFFECTING UNSP. SIDE
3429	HEMIPLEGIA, UNSPECIFIED
34290	HEMIPLEGIA, UNSP., AFFECTING UNSP. SIDE
34291	HEMIPLEGIA, UNSP., AFFECTING DOMINANT SIDE
34292	HEMIPLEGIA, UNSP., AFFECTING NONDOMINANT SIDE
3430	CONGENITAL DIPLEGIA

3431	CONGENITAL HEMIPLEGIA
3432	CONGENITAL QUADRIPLEGIA
3439	INFANTILE CEREBRAL PALSY, UNSPECIFIED
34400	QUADRIPLEGIA, UNSPECIFIED
3441	PARAPLEGIA
3442	DIPLEGIA OF UPPER LIMBS
34430	MONOPLEGIA OF LOWER LIMB, AFFECTING UNSP. SIDE
34440	MONOPLEGIA OF UPPER LIMB, AFFECTING UNSP. SIDE
34489	OTHER SPECIFIED PARALYTIC SYNDROME
3449	PARALYSIS, UNSPECIFIED
3481	ANOXIC BRAIN DAMAGE
3526	MULTIPLE CRANIAL NERVE PALSIES
43820	HEMIPLEGIA AFFECTING UNSP. SIDE
7814	TRANSIENT PARALYSIS OF LIMB
2930	ACUTE DELIRIUM
2930	DELIRIUM DUE TO CONDITIONS CLASSIFIED ELSEWHERE
29384	ANXIETY DISORDER IN CONDITIONS CLASSIFIED ELSEWHERE
2940	AMNESTIC DISORDER IN CONDITIONS CLASSIFIED ELSEWHERE
2949	UNSPECIFIED PERSISTENT MENTAL DISORDERS DUE TO COND.CLASS.ELSEWH.
29530	PARANOID TYPE SCHIZOPHRENIA, UNSPECIFIED STATE
29570	SCHIZOAFFECTIVE DISORDER SCHIZOPHRENIA, UNSPECIFIED STATE
29580	OTHER SPECIFIED TYPES OF SCHIZOPHRENIA, UNSPECIFIED STATE
29590	UNSPECIFIED TYPE SCHIZOPHRENIA, UNSPECIFIED STATE
29600	BIPOLAR I DISORDER, SINGLE MANIC EPISODE, UNSPECIFIED DEGREE
29620	MAJOR DEPRESSIVE AFFECTIVE DISORDER,SINGLE EPISODE,UNSP.DEGREE
29680	BIPOLAR DISORDER, UNSPECIFIED
29690	UNSPECIFIED EPISODIC MOOD DISORDER
29699	OTHER SPECIFIED AFFECTIVE PSYCHOSES
2971	DELUSIONAL DISORDER
2979	UNSPECIFIED PARANOID STATE
2981	EXCITATIVE TYPE PSYCHOSIS
2983	ACUTE PARANOID REACTION
2989	UNSPECIFIED PSYCHOSIS
30000	ANXIETY STATE, UNSPECIFIED
30001	PANIC DISORDER WITHOUT AGORAPHOBIA
30009	OTHER ANXIETY STATES
30010	HYSTERIA, UNSPECIFIED
30011	CONVERSION DISORDER
30029	OTHER ISOLATED OR SIMPLE PHOBIAS
3003	OBSESSIVE-COMPULSIVE DISORDERS
3004	DYSTHYMIC DISORDER
3004	NEUROTIC DEPRESSION
3009	UNSPECIFIED NONPSYCHOTIC MENTAL DISORDER
30183	BORDERLINE PERSONALITY
30183	BORDERLINE PERSONALITY DISORDER
3019	UNSPECIFIED PERSONALITY DISORDER
3026	DISORDERS OF PSYCHOSEXUAL IDENTITY
30302	AC.ALCOHOLIC INTOXIC.IN ALCOHOLISM,EPISODIC DRINKING BEHAVIOR
30400	OPIOID TYPE DEPENDENCE, UNSPECIFIED USE
30430	CANNABIS DEPENDENCE, UNSPECIFIED USE

30432	CANNABIS DEPENDENCE, EPISODIC USE
30500	ALCOHOL ABUSE, UNSPECIFIED DRINKING BEHAVIOR
30501	ALCOHOL ABUSE, CONTINUOUS DRINKING BEHAVIOR
30502	ALCOHOL ABUSE, EPISODIC DRINKING BEHAVIOR
3051	TOBACCO USE DISORDER (TOBACCO DEPENDENCE)
30591	OTHER, MIXED, OR UNSPECIFIED DRUG ABUSE, CONTINUOUS USE
3061	RESPIRATORY MALFUNCTION ARISING FROM MENTAL FACTORS
3062	CARDIOVASCULAR MALFUNCTION ARISING FROM MENTAL FACTORS
3068	OTHER SPECIFIED PSYCHOPHYSIOLOGICAL MALFUNCTION
3069	UNSPECIFIED PSYCHOPHYSIOLOGICAL MALFUNCTION
3070	ADULT ONSET FLUENCY DISORDER
3070	STAMMERING AND STUTTERING
3070	STUTTERING
30720	TIC DISORDER, UNSPECIFIED
30722	CHRONIC MOTOR OR VOCAL TIC DISORDER
30723	TOURETTE'S DISORDER
30752	PICA
3080	PREDOMINANT DISTURBANCE OF EMOTIONS
3089	UNSPECIFIED ACUTE REACTION TO STRESS
309	ADJUSTMENT REACTION
3090	ADJUSTMENT DISORDER WITH DEPRESSED MOOD
30924	ADJUSTMENT DISORDER WITH ANXIETY
3094	ADJUSTMENT DISOR.WITH MIXED DISTURB.OF EMOTIONS AND CONDUCT
30981	POSTTRAUMATIC STRESS DISORDER
3099	UNSPECIFIED ADJUSTMENT REACTION
311	DEPRESSIVE DISORDER, NOT ELSEWHERE CLASSIFIED
31210	UNDERSOCIALIZED CONDUCT DISORDER,UNAGGRESSIVE TYPE,UNSPECIFIED
31239	OTHER DISORDERS OF IMPULSE CONTROL
3129	UNSPECIFIED DISTURBANCE OF CONDUCT
31389	OTHER EMOTIONAL DISTURBANCES OF CHILDHOOD OR ADOLESCENCE
3139	UNSPECIFIED EMOTIONAL DISTURBANCE OF CHILDHOOD OR ADOLESCENCE
316	PSYCHIC FACTORS ASSOCIATED WITH DISEASES CLASSIFIED ELSEWHERE
7801	HALLUCINATIONS
7803	CONVULSIONS
31400	ATTENTION DEFICIT DISORDER WITHOUT HYPERACTIVITY
31401	ATTENTION DEFICIT DISORDER WITH HYPERACTIVITY
3142	HYPERKINETIC CONDUCT DISORDER OF CHILDHOOD
3149	UNSPECIFIED HYPERKINETIC SYNDROME OF CHILDHOOD
3152	OTHER SPECIFIC DEVELOPMENTAL LEARNING DIFFICULTIES
31531	EXPRESSIVE LANGUAGE DISORDER
31534	SPEECH AND LANGUAGE DEVELOPMENTAL DELAY DUE TO HEARING LOSS
31539	OTHER DEVELOPMENTAL SPEECH DISORDER
3154	DEVELOPMENTAL COORDINATION DISORDER
3158	OTHER SPECIFIED DELAYS IN DEVELOPMENT
3159	UNSPECIFIED DELAY IN DEVELOPMENT
317	MILD INTELLECUTAL DISABILITIES
317	MILD MENTAL RETARDATION
319	UNSPECIFIED INTELLECTUAL DISABILITIES
319	UNSPECIFIED MENTAL RETARDATION
33183	MILD COGNITIVE IMPAIRMENT, SO STATED

7834	LACK OF EXPECTED NORMAL PHYSIOLOGICAL DEVELOPMENT
7834	LACK OF EXPECTED NORMAL PHYSIOLOGICAL DEVELOPMENT IN CHILDHOOD
78340	LACK OF NORMAL PHYSIOLOGICAL DEVELOPMENT, UNSPECIFIED
330	CEREBRAL DEGENERATIONS USUALLY MANIFEST IN CHILDHOOD
3300	LEUKODYSTROPHY
3308	OTHER SPECIFIED CEREBRAL DEGENERATIONS IN CHILDHOOD
3313	COMMUNICATING HYDROCEPHALUS
3314	OBSTRUCTIVE HYDROCEPHALUS
33189	OTHER CEREBRAL DEGENERATION
3319	CEREBRAL DEGENERATION, UNSPECIFIED
3348	OTHER SPINOCEREBELLAR DISEASES
335	ANTERIOR HORN CELL DISEASE
3350	WERDNIG-HOFFMANN DISEASE
33510	SPINAL MUSCULAR ATROPHY, UNSPECIFIED
33522	PROGRESSIVE BULBAR PALSY
33523	PSEUDOBULBAR PALSY
3360	SYRINGOMYELIA AND SYRINGOBULBIA
340	MULTIPLE SCLEROSIS
34120	ACUTE (TRANSVERSE) MYELITIS NOS
3419	DEMYELINATING DISEASE OF CENTRAL NERVOUS SYSTEM, UNSPECIFIED
3480	CEREBRAL CYSTS
3590	CONGENITAL HEREDITARY MUSCULAR DYSTROPHY
3591	HEREDITARY PROGRESSIVE MUSCULAR DYSTROPHY
34600	MIGRAINE WITH AURA WITHOUT MENTION OF INTRACTABLE MIGRAINE,WITHOU T MENTION
34601	MIGRAINE WITH AURA,SO STATED,WITHOUT MENTION OF STATU.MIGRAINOSUS
34620	VARIANTS OF MIGRAINE, WITHOUT INTRACTABLE MIGRAINE
34630	HEMIPLEGIC MIGRAINE WITHOUT MENTION OF INTRACTABLE MIGRAINE, WITH OUT MENTIOI
34670	CHRONIC MIGRAINE WITHOUT AURA WITHOUT MENTION OF INTRACTABLE MIGR AINE, WITH
3469	MIGRAINE, UNSPECIFIED
34690	MIGRAINE, UNSPECIFIED, WITHOUT INTRACTABEL MIGRAINE
34690	MIGRAINE, UNSPECIFIED, WITHOUT MENTION OF INTRACTABLE MIGRAINE WI THOUT MENTIC
30789	OTHER PSYCHALGIA
7802	SYNCOPE AND COLLAPSE
78093	MEMORY LOSS
7843	APHASIA
	**Infectious diagnoses**
5901	ACUTE PYELONEPHRITIS
5990	URINARY TRACT INFECTION, SITE NOT SPECIFIED
59010	AC.PYELONEPHRITIS WITHOUT LESION OF RENAL MEDULLARY NECROSIS
59080	PYELONEPHRITIS, UNSPECIFIED
5950	ACUTE CYSTITIS
59581	CYSTITIS CYSTICA
59589	OTHER SPECIFIED TYPES OF CYSTITIS
5959	CYSTITIS, UNSPECIFIED
5970	URETHRAL ABSCESS
59780	URETHRITIS, UNSPECIFIED
59789	OTHER URETHRITIS
0030	SALMONELLA GASTROENTERITIS
0039	SALMONELLA INFECTION, UNSPECIFIED
0040	SHIGELLA DYSENTERIAE

0041	SHIGELLA FLEXNERI
0042	SHIGELLA BOYDII
0043	SHIGELLA SONNEI
0048	OTHER SPECIFIED SHIGELLA INFECTIONS
0049	SHIGELLOSIS, UNSPECIFIED
0051	BOTULISM
0059	FOOD POISONING, UNSPECIFIED
008	INTESTINAL INFECTIONS DUE TO OTHER ORGANISMS
0084	INTESTINAL INFECTION DUE TO OTHER SPECIFIED BACTERIA
00842	INTESTINAL INFEC. DUE TO PSEUDOMONAS
00843	INTESTINAL INFEC. DUE TO CAMPYLOBACTER
00845	INTESTINAL INFEC. DUE TO CLOSTRIDIUM DIFFICILE
0085	BACTERIAL ENTERITIS, UNSPECIFIED
00861	ENTERITIS DUE TO ROTAVIRUS
00862	ENTERITIS DUE TO ADENOVIRUS
00865	ENTERITIS DUE TO CALICIVIRUS
00867	ENTERITIS DUE TO ENTEROVIRUS, N.E.C.
00869	OTHER VIRAL ENTERITIS
0088	INTESTINAL INFECTION DUE TO OTHER ORGANISM,NOT ELSEW.CLASS.
0090	INFECTIOUS COLITIS, ENTERITIS, & GASTROENTERITIS
0090	INFECTIOUS COLITIS, ENTERITIS, AND GASTROENTERITIS
0091	COLITIS,ENTERITIS,GASTROENTERITIS OF PRESUMED INF. ORIGIN
0092	INFECTIOUS DIARRHEA
5902	RENAL AND PERINEPHRIC ABSCESS
0022	PARATYPHOID FEVER B
0023	PARATYPHOID FEVER C
320	BACTERIAL MENINGITIS
3200	HEMOPHILUS MENINGITIS
3201	PNEUMOCOCCAL MENINGITIS
3202	STREPTOCOCCAL MENINGITIS
3203	STAPHYLOCOCCAL MENINGITIS
3207	MENINGITIS IN OTHER BACTERIAL DISEASES CLASSIFIED ELSEWHERE
3208	MENINGITIS DUE TO OTHER SPECIFIED BACTERIA
32082	MENINGITIS DUE TO GRAM-NEGATIVE
32089	MENINGITIS DUE TO OTHER SPECIFIED BACTERIA
3209	MENINGITIS DUE TO UNSPECIFIED BACTERIUM
3240	INTRACRANIAL ABSCESS
3241	INTRASPINAL ABSCESS
3249	INTRACRANIAL AND INTRASPINAL ABSCESS OF UNSPECIFIED SITE
325	PHLEBITIS AND THROMBOPHLEBITIS OF INTRACRANIAL VENOUS SINUSES
326	LATE EFFECTS OF INTRACRANIAL ABSCESS OR PYOGENIC INFECTION
0031	SALMONELLA SEPTICEMIA
00321	SALMONELLA MENINGITIS
00323	SALMONELLA ARTHRITIS
00329	OTHER LOCALIZED SALMONELLA INFECTIONS
0038	OTHER SPECIFIED SALMONELLA INFECTIONS
04186	HELICOBACTER PYLORI (H. PYLORI) INFECTION
1274	ENTEROBIASIS
1278	MIXED INTESTINAL HELMINTHIASIS
1279	INTESTINAL HELMINTHIASIS, UNSPECIFIED

1289	HELMINTH INFECTION, UNSPECIFIED
01090	PRIM. TB. INFEC., UNSP.TYPE, UNSP.EXAMINATION
01120	TB. OF LUNG + CAVITATION, UNSP. EXAMINATION
0119	UNSPECIFIED PULMONARY TUBERCULOSIS
01190	UNSP. PULMONARY TB., UNSP. EXAMINATION
01194	UNSP. PULMONARY TB., FOUND BY BACTERIAL CULTURE
01304	TB. MENINGITIS, FOUND BY BACTERIAL CULTURE
01311	TUBERCULOMA OF MENINGES, BACT/HISTOL. EXAM. NOT DONE
01394	UNSP. TB. OF C.N.S. FOUND BY BACTERIAL CULTURE
01404	TB. PERITONITIS, FOUND BY BACTERIAL CULTURE
01485	OTHER TB. INTESTINES, CONFIRMED HISTOLOGICALLY
0073	INTESTINAL TRICHOMONIASIS
0074	CRYPTOSPORIDIOSIS
0075	CYCLOSPORIASIS
0082	INTESTINAL INFEC. DUE TO AEROBACTER AEROGENES
00841	INTESTINAL INFEC. DUE TO STAPHYLOCOCCUS
0205	PNEUMONIC PLAGUE, UNSPECIFIED
0209	PLAGUE, UNSPECIFIED
022	ANTHRAX
0810	MURINE (ENDEMIC) TYPHUS
0820	SPOTTED FEVERS
0830	Q FEVER
0838	OTHER SPECIFIED RICKETTSIOSES
0839	RICKETTSIOSIS, UNSPECIFIED
0846	MALARIA, UNSPECIFIED
0859	LEISHMANIASIS, UNSPECIFIED
0879	RELAPSING FEVER, UNSPECIFIED
08881	LYME DISEASE (ERYTHEMA CHRONICUM MIGRANS)
08882	BABESIOSIS
0909	CONGENITAL SYPHILIS, UNSPECIFIED
0940	TABES DORSALIS
0980	GONOCOCCAL INFEC.,ACUTE, OF LOWER GENITOURINARY TRACT
09840	GONOCOCCAL CONJUNCTIVITIS (NEONATORUM)
09882	GONOCOCCAL MENINGITIS
09886	GONOCOCCAL PERITONITIS
1000	LEPTOSPIROSIS ICTEROHEMORRHAGICA
1229	ECHINOCOCCOSIS, OTHER AND UNSPECIFIED
1270	ASCARIASIS
1363	PNEUMOCYSTOSIS
1369	UNSP. INFECTIOUS & PARASITIC DISEASES
1369	UNSPECIFIED INFECTIOUS AND PARASITIC DISEASES
0239	BRUCELLOSIS, UNSPECIFIED
0269	UNSPECIFIED RAT-BITE FEVER
0270	LISTERIOSIS
0312	DISSEMINATED DISEASE DUE TO OTHER MYCOBACTERIA
0319	UNSPECIFIED DISEASES DUE TO MYCOBACTERIA
03283	DIPHTHERITIC PERITONITIS
0068	AMEBIC INFECTION OF OTHER SITES
0069	AMEBIASIS, UNSPECIFIED
0070	BALANTIDIASIS

0071	GIARDIASIS
0078	OTHER SPECIFIED PROTOZOAL INTESTINAL DISEASES
0079	UNSPECIFIED PROTOZOAL INTESTINAL DISEASE
129	INTESTINAL PARASITISM, UNSPECIFIED
0418	OTHER SPEC.BACTERIAL INF;IN CONDIT.CLASS.ELSEWHERE,UNSP.SITE
04189	OTHER SPECIFIED BACTERIA INFECTION
0419	UNSP. BACTERIAL INFECTION, UNSP. SITE
0419	UNSPEC.BACTERIAL INF;IN CONDIT. CLASSIF.ELSEWHERE,UNSP.SITE
0330	WHOOPING COUGH DUE TO BORDETELLA PERTUSSIS (B. PERTUSSIS)
0331	WHOOPING COUGH DUE TO BORDETELLA PARAPERTUSSIS
0339	WHOOPING COUGH, UNSPECIFIED ORGANISM
034	STREPTOCOCCAL SORE THROAT AND SCARLET FEVER
0340	STREPTOCOCCAL SORE THROAT
0341	SCARLET FEVER
035	ERYSIPELAS
0410	STREPTOCOCCUS INFECT.IN CONDITION CLASSIF.ELSEWHERE;UNSP.SITE
04100	STREPTOCOCCUS INFEC., UNSP.
04101	STREPTOCOCCUS INFEC., GROUP A
04101	STREPTOCOCCUS INFEC., GROUP A (ADDITIONAL CODE)
04102	STREPTOCOCCUS INFEC., GROUP B
04103	STREPTOCOCCUS INFEC., GROUP C
04104	STREPTOCOCCUS INFEC., GROUP D (ENTEROCOCCUS)
04105	STREPTOCOCCUS INFEC., GROUP G
04109	OTHER STREPTOCOCCUS INFEC.
0411	STAPHYLOCOCCUS INFECT.IN CONDIT.CLASSIF.ELSEWHERE,UNSP.SITE
0411	STAPHYLOCOCCUS INFECTION, UNSP. SITE
04110	STAPHYLOCOCCUS INFECTION, UNSP.
04111	METHICILLIN SUSCEPTIBLE STAPHYLOCOCCUS AUREUS
04111	STAPHYLOCOCCUS AUREUS INFEC.
04111	STAPHYLOCOCCUS AUREUS INFEC. (ADDITIONAL CODE)
04112	METHICILLIN RESISTANT STAPHYLOCOCCUS AUREUS
04119	OTHER STAPHYLOCOCCUS INFECTION
0412	PNEUMOCOCCUS INFECT.IN CONDITION CLASSIF.ELSEWHERE;UNSP.SITE
0412	PNEUMOCOCCUS INFECTION, UNSP. SITE
0360	MENINGOCOCCAL MENINGITIS
0362	MENINGOCOCCEMIA
03682	MENINGOCOCCAL ARTHROPATHY
03689	OTHER SPECIFIED MENINGOCOCCAL INFECTIONS
0369	MENINGOCOCCAL INFECTION, UNSPECIFIED
0380	STREPTOCOCCAL SEPTICEMIA
0381	STAPHYLOCOCCAL SEPTICEMIA
03810	STAPHYLOCOCCAL SPETICEMIA, UNSP.
03811	METHICILLIN SUSCEPTIBLE STAPHYLOCOCCUS AUREUS SEPTICEMIA
03811	STAPHYLOCOCCUS AUREUS SEPTICEMIA
03812	METHICILLIN RESISTANT STAPHYLOCOCCUS AUREUS SEPTICEMIA
03819	OTHER STAPHYLOCOCCAL SEPTICEMIA
0382	PNEUMOCOCCAL SEPTICEMIA
0383	SEPTICEMIA DUE TO ANAEROBES
03840	SEPTICEMIA DUE TO GRAM-NEGATIVE ORGANISM, UNSPECIFIED
03841	SEPTICEMIA DUE TO HEMOPHILUS INFLUENZAE (H. INFLUENZAE)

03842	SEPTICEMIA DUE TO ESCHERICHIA COLI (E. COLI)
03843	SEPTICEMIA DUE TO PSEUDOMONAS
03849	OTHER SEPTICEMIA DUE TO GRAM-NEGATIVE ORGANISMS
038491	KLEBSIELLA SEPTICEMIA
0388	OTHER SPECIFIED SEPTICEMIAS
0388 1	CANDIDA SEPTICEMIA
0388 2	ENTEROCOCCUS SEPTICEMIA
0388 3	ACITINOBACTER SEPTICEMIA
0389	UNSPECIFIED SEPTICEMIA
0413	FRIEDLANDER'S BACILLUS, UNSP. SITE
0413	FRIEDLANDER'S BACILLUS;CONDITION CLASSIF.ELSEWHERE,UNSP.SITE
0413	KLEBSIELLA PNEUMONIAE
0414	ESCHERICHIA COLI (E. COLI), UNSP. SITE
0414	ESCHERICHIA COLI (E. COLI), UNSP. SITE (ADDITIONAL CODE)
0414	ESCHERICHIA COLI(E. COLI),CONDITI.CLASSIF.ELSEWHERE,UNSP.SITE
0415	HEMOPHILUS INFLUENZAE IN CONDIT.CLASSIF.ELSEWHERE,UNSP.SITE
0415	HEMOPHILUS INFLUENZAE, UNSP. SITE (H.INFLUENZAE)
0416	PROTEUS (MIRABILIS,MORGANII), UNSP. SITE
0416	PROTEUS(MIRABILIS,MORGANII)CONDIT,CLASSIF.ELSEWHERE,UNSP.SITE
0417	PSEUDOMONAS INFEC., UNSP. SITE
0417	PSEUDOMONAS INFECT.IN CONDIT.CLASSIF.ELSEWHERE,UNSPEC.SITE
04185	OTHER GRAM-NEGATIVE ORGANISMS INFECTION
042	HUMAN IMMUNODEFIC. VIRUS (HIV) DIS. /AIDS
0470	MENINGITIS DUE TO COXSACKIE VIRUS
0471	MENINGITIS DUE TO ECHO VIRUS
0478	OTHER SPECIFIED VIRAL MENINGITIS
0479	UNSPECIFIED VIRAL MENINGITIS
048	OTHER ENTEROVIRUS DISEASES OF CENTRAL NERVOUS SYSTEM
0491	NON-ARTHOPOD-BORNE MENINGITIS DUE TO ADENOVIRUS
0499	UNSP.NON-ARTHROPOD-BORNE VIRAL DIS.OF CENTRAL NERVOUS SYSTEM
3236	POSTINFECTIOUS ENCEPHALITIS
32361	INFECTIOUS ACUTE DISSEMINATED ENCEPHALOMYELITIS (ADEM)
0521	VARICELLA (HEMORRHAGIC) PNEUMONITIS
0527	CHICKENPOX WITH OTHER SPECIFIED COMPLICATIONS
0528	CHICKENPOX WITH UNSPECIFIED COMPLICATION
0529	VARICELLA WITHOUT MENTION OF COMPLICATION
0540	ECZEMA HERPETICUM
05410	GENITAL HERPES, UNSPECIFIED
0542	HERPETIC GINGIVOSTOMATITIS
0543	HERPETIC MENINGOENCEPHALITIS
05440	HERPES SIMPLEX + UNSP. OPHTHALMIC COMPLICATION
05441	HERPES SIMPLEX DERMATITIS OF EYELID
05443	HERPES SIMPLEX DISCIFORM KERATITIS
05449	HERPES SIMPLEX WITH OTHER OPHTHALMIC COMPLICATIONS
0546	HERPETIC WHITLOW
05472	HERPES SIMPLEX MENINGITIS
05479	HERPES SIMPLEX + OTHER SPEC. COMPLICATIONS
0549	HERPES SIMPLEX WITHOUT MENTION OF COMPLICATION
05829	OTHER HUMAN HERPESVIRUS ENCEPHALITIS
05311	GENICULATE HERPES ZOSTER

05319	HERPES ZOSTER + OTHER NERVOUS SYSTEM COMPLICATIONS
05320	HERPES ZOSTER DERMATITIS OF EYELID
05329	HERPES ZOSTER + OTHER OPHTHALMIC COMPLICATIONS
0539	HERPES ZOSTER WITHOUT MENTION OF COMPLICATION
0559	MEASLES WITHOUT MENTION OF COMPLICATION
0569	RUBELLA WITHOUT MENTION OF COMPLICATION
0570	ERYTHEMA INFECTIOSUM (FIFTH DISEASE)
0578	OTHER SPECIFIED VIRAL EXANTHEMATA
0579	VIRAL EXANTHEM, UNSPECIFIED
05810	ROSEOLA INFANTUM, UNSPECIFIED
0729	MUMPS WITHOUT MENTION OF COMPLICATION
0701	VIRAL HEPATITIS A WITHOUT HEPATIC COMA
0701	VIRAL HEPATITIS A WITHOUT MENTION OF HEPATIC COMA
07030	VIRAL HEPATITIS B WITHOUT HEPATIC COMA & HEPATITIS DELTA -92
07030	VIRAL HEPATITIS B WITHOUT HEPATIC COMA,AC/UNSP.WITHOUT HEP. DELTA
07032	VIRAL HEPATITS B WITHOUT HEPATIC COMA,CHR. WITHOUT HEPATITIS DELT
0709	UNSP. VIRAL HEPATITIS WITHOUT HEPATIC COMA
0709	UNSPECIFIED VIRAL HEPATITIS WITHOUT MENTION OF HEPATIC COMA
075	INFECTIOUS MONONUCLEOSIS
0773	OTHER ADENOVIRAL CONJUNCTIVITIS
0774	EPIDEMIC HEMORRHAGIC CONJUNCTIVITIS
0779	UNSPEC.DISEASES OF CONJUNCTIVA DUE TO VIRUSES AND CHLAMYDIAE
07799	UNSP. DIS. OF CONJUNCTIVA DUE TO VIRUSES
0780	MOLLUSCUM CONTAGIOSUM
0781	VIRAL WARTS
07810	VIRAL WARTS, UNSPECIFIED
07811	CONDYLOMA ACUMINATUM
07812	PLANTAR WART
07819	OTHER SPECIFIED VIRAL WARTS
0785	CYTOMEGALIC INCLUSION DISEASE
0785	CYTOMEGALOVIRAL DISEASE
07889	OTHER SPECIFIED DISEASES DUE TO VIRUSES
07889	OTHER SPECIFIED DISEASES DUE TO VIRUSES AND CHLAMYDIAE
0790	ADENOVIRUS INF.IN CONDITIONS CLASSIF.ELSEWHERE,UNSP.SITE
0790	ADENOVIRUS INFECTION, UNSP. SITE
0791	ECHO VIRUS INFECTION, UNSP. SITE
0792	COXSACKIE VIRUS INFECTION, UNSP. SITE
0793	RHINOVIRUS INFECTION, UNSP. SITE
07950	RETROVIRUS, UNSP.,UNSP. SITE
07959	OTHER SPECIFIED RETROVIRUS
0798	OTHER SPECIFIED VIRAL INFECTION CLASSIF.ELSEWHERE,UNSP.SITE
07989	OTHER SPEC. VIRAL INFECTION
0799	UNSP. VIRAL & CHLANYDIAL INFECTION
0799	UNSP.VIRAL INFECT.IN CONDITIONS CLASSIF.ELSEWHERE,UNSP.SITE
07999	UNSP. VIRAL INFECTION
07999	UNSP. VIRAL INFECTION (ADDITIONAL CODE)
0796	RESPIRATORY SYNCYTIAL VIRUS (RSV)
1100	DERMATOPHYTOSIS OF SCALP AND BEARD
1101	DERMATOPHYTOSIS OF NAIL
1103	DERMATOPHYTOSIS OF GROIN AND PERIANAL AREA

1104	DERMATOPHYTOSIS OF FOOT
1105	DERMATOPHYTOSIS OF THE BODY
1109	DERMATOPHYTOSIS OF UNSPECIFIED SITE
1110	PITYRIASIS VERSICOLOR
1118	OTHER SPECIFIED DERMATOMYCOSES
1119	DERMATOMYCOSIS, UNSPECIFIED
1120	CANDIDIASIS OF MOUTH
1121	CANDIDIASIS OF VULVA AND VAGINA
38001	ACUTE PERICHONDRITIS OF PINNA
38002	CHRONIC PERICHONDRITIS OF PINNA
38003	CHONDRITIS OF PINNA
38010	INFECTIVE OTITIS EXTERNA, UNSPECIFIED
38015	CHRONIC MYCOTIC OTITIS EXTERNA
38022	OTHER ACUTE OTITIS EXTERNA
38023	OTHER CHRONIC OTITIS EXTERNA
38100	ACUTE NONSUPPURATIVE OTITIS MEDIA, UNSPECIFIED
38101	ACUTE SEROUS OTITIS MEDIA
38104	ACUTE ALLERGIC SEROUS OTITIS MEDIA
38110	CHRONIC SEROUS OTITIS MEDIA, SIMPLE OR UNSPECIFIED
38119	OTHER CHRONIC SEROUS OTITIS MEDIA
38129	OTHER CHRONIC MUCOID OTITIS MEDIA
3813	OTHER AND UNSPECIFIED CHRONIC NONSUPPURATIVE OTITIS MEDIA
3814	NONSUPPURATIVE OTITIS MEDIA, NOT SPECIFIED AS ACUTE OR CHRONIC
3819	UNSPECIFIED EUSTACHIAN TUBE DISORDER
382	SUPPURATIVE AND UNSPECIFIED OTITIS MEDIA
3820	ACUTE SUPPURATIVE OTITIS MEDIA
38200	AC.SUPPURAT.OTITIS MEDIA WITHOUT SPONTAN.RUPTURE OF EARDRUM
38201	ACUTE SUPPURAT.OTITIS MEDIA WITH SPONTAN.RUPTURE OF EARDRUM
3821	CHRONIC TUBOTYMPANIC SUPPURATIVE OTITIS MEDIA
3823	UNSPECIFIED CHRONIC SUPPURATIVE OTITIS MEDIA
3824	UNSPECIFIED SUPPURATIVE OTITIS MEDIA
3829	UNSPECIFIED OTITIS MEDIA
38300	ACUTE MASTOIDITIS WITHOUT COMPLICATIONS
38301	SUBPERIOSTEAL ABSCESS OF MASTOID
3831	CHRONIC MASTOIDITIS
38330	POSTMASTOIDECTOMY COMPLICATION, UNSPECIFIED
38389	OTHER DISORDERS OF MASTOID
3839	UNSPECIFIED MASTOIDITIS
38400	ACUTE MYRINGITIS, UNSPECIFIED
38401	BULLOUS MYRINGITIS
3841	CHRONIC MYRINGITIS WITHOUT MENTION OF OTITIS MEDIA
38420	PERFORATION OF TYMPANIC MEMBRANE, UNSPECIFIED
38482	ATROPHIC NONFLACCID TYMPANIC MEMBRANE
3849	UNSPECIFIED DISORDER OF TYMPANIC MEMBRANE
38500	TYMPANOSCLEROSIS, UNSPECIFIED AS TO INVOLVEMENT
38510	ADHESIVE MIDDLE EAR DISEASE, UNSPECIFIED AS TO INVOLVEMENT
38530	CHOLESTEATOMA, UNSPECIFIED
38531	CHOLESTEATOMA OF ATTIC
38532	CHOLESTEATOMA OF MIDDLE EAR
38630	LABYRINTHITIS, UNSPECIFIED

38635	VIRAL LABYRINTHITIS
460	ACUTE NASOPHARYNGITIS (COMMON COLD)
4610	ACUTE MAXILLARY SINUSITIS
4611	ACUTE FRONTAL SINUSITIS
4612	ACUTE ETHMOIDAL SINUSITIS
4613	ACUTE SPHENOIDAL SINUSITIS
4618	OTHER ACUTE SINUSITIS
4619	ACUTE SINUSITIS, UNSPECIFIED
462	ACUTE PHARYNGITIS
463	ACUTE TONSILLITIS
464	ACUTE LARYNGITIS AND TRACHEITIS
4640	ACUTE LARYNGITIS
46400	ACUTE LARYNGITIS WITHOUT MENTION OF OBSTRUCTION
46410	ACUTE TRACHEITIS WITHOUT MENTION OF OBSTRUCTION
46420	ACUTE LARYNGOTRACHEITIS WITHOUT MENTION OF OBSTRUCTION
46430	ACUTE EPIGLOTTITIS WITHOUT MENTION OF OBSTRUCTION
4644	CROUP
46450	SUPRAGLOTTITIS WITHOUT MENTION OF OBSTRUCTION
465	ACUTE UPPER RESPIRATORY INFECTIONS OF MULTIPLE OR UNSP.SITES
4650	ACUTE LARYNGOPHARYNGITIS
4658	ACUTE UPPER RESPIRATORY INFECTIONS OF OTHER MULTIPLE SITES
4659	ACUTE UPPER RESPIRATORY INFECTIONS OF UNSPECIFIED SITE
4720	CHRONIC RHINITIS
4730	CHRONIC MAXILLARY SINUSITIS
4731	CHRONIC FRONTAL SINUSITIS
4732	CHRONIC ETHMOIDAL SINUSITIS
4733	CHRONIC SPHENOIDAL SINUSITIS
4738	OTHER CHRONIC SINUSITIS
4739	UNSPECIFIED SINUSITIS (CHRONIC)
47400	CHRONIC TONSILLITIS
47401	CHRONIC ADENOIDITIS
4741	HYPERTROPHY OF TONSILS AND ADENOIDS
47410	HYPERTROPHY OF TONSIL WITH ADENOIDS
47411	HYPERTROPHY OF TONSILS ALONE
47412	HYPERTROPHY OF ADENOIDS ALONE
4742	ADENOID VEGETATIONS
4748	OTHER CHRONIC DISEASE OF TONSILS AND ADENOIDS
4749	UNSPECIFIED CHRONIC DISEASE OF TONSILS AND ADENOIDS
475	PERITONSILLAR ABSCESS
47822	PARAPHARYNGEAL ABSCESS
47824	RETROPHARYNGEAL ABSCESS
4870	INFLUENZA WITH PNEUMONIA
4871	INFLUENZA WITH OTHER RESPIRATORY MANIFESTATIONS
4878	INFLUENZA WITH OTHER MANIFESTATIONS
4880	INFLUENZA DUE TO IDENTIFIED AVIAN INFLUENZA VIRUS
4881	INFLUENZA DUE TO IDENTIFIED NOVEL H1N1 INFLUENZA VIRUS
466	ACUTE BRONCHITIS AND BRONCHIOLITIS
4660	ACUTE BRONCHITIS
4661	ACUTE BRONCHIOLITIS
46611	AC. BRONCHIOLITIS DUE TO RESPIRATORY SYNCYTIAL VIRUS (RSV)

46619	AC. BRONCHIOLITIS DUE TO OTHER INFECTIOUS ORGANISMS
480	VIRAL PNEUMONIA
4800	PNEUMONIA DUE TO ADENOVIRUS
4801	PNEUMONIA DUE TO RESPIRATORY SYNCYTIAL VIRUS
4802	PNEUMONIA DUE TO PARAINFLUENZA VIRUS
4808	PNEUMONIA DUE TO OTHER VIRUS NOT ELSEWHERE CLASSIFIED
4809	VIRAL PNEUMONIA, UNSPECIFIED
4841	PNEUMONIA IN CYTOMEGALIC INCLUSION DISEASE
490	BRONCHITIS, NOT SPECIFIED AS ACUTE OR CHRONIC
49121	OBSTRUCTIVE CHR. BRONCHITIS WITH(ACUTE)EXACERBATION
4918	OTHER CHRONIC BRONCHITIS
481	PNEUMOCOCCAL PNEUMONIA
481	PNEUMOCOCCAL PNEUMONIA (STREPTOCOCUUS PNEUMONIAE PNEUMONIA)
4820	PNEUMONIA DUE TO KLEBSIELLA PNEUMONIAE
4821	PNEUMONIA DUE TO PSEUDOMONAS
4822	PNEUMONIA DUE TO HEMOPHILUS INFLUENZAE (H. INFLUENZAE)
4823	PNEUMONIA DUE TO STREPTOCOCCUS
48230	PNEUMONIA DUE TO STREPTOCOCCUS, UNSPECIFIED
48231	PNEUMONIA DUE TO STREPTOCOCCUS, GROUP A
48241	PNEUMONIA DUE TO STAPHYLOCOCCUS AUREUS
4829	BACTERIAL PNEUMONIA, UNSPECIFIED
483	PNEUMONIA DUE TO OTHER SPECIFIED ORGANISM
4830	PNEUMONIA DUE TO MYCOPLASMA PNEUMONIAE
4831	PNEUMONIA DEU TO CHLAMYDIA
4838	PNEUMONIA DUE TO OTHER SPECIFIED ORGANISM
04181	OTHER MYCOPLASMA INFECTION
04181	OTHER MYCOPLASMA INFECTION (ADDITIONAL CODE)
04183	OTHER CLOSTRIDIUM PERFRINGENS INFECTION
04184	OTHER ANAEROBES INFECTION
0783	CAT-SCRATCH DISEASE
0784	FOOT AND MOUTH DISEASE
1330	SCABIES
**Neuoplasm diagnoses**	
1409	MALIGNANT NEOPLASM OF LIP, UNSP., VERMILION BORDER
1420	MALIGNANT NEOPLASM OF PAROTID GLAND
1450	MALIGNANT NEOPLASM OF CHEEK MUCOSA
1479	MALIGNANT NEOPLASM OF NASOPHARYNX, UNSPECIFIED
1502	MALIGNANT NEOPLASM OF ABDOMINAL ESOPHAGUS
1519	MALIGNANT NEOPLASM OF STOMACH, UNSPECIFIED
1540	MALIGNANT NEOPLASM OF RECTOSIGMOID JUNCTION
1541	MALIGNANT NEOPLASM OF RECTUM
1550	MALIGNANT NEOPLASM OF LIVER, PRIMARY
1570	MALIGNANT NEOPLASM OF HEAD OF PANCREAS
1579	MALIGNANT NEOPLASM OF PANCREAS, PART UNSPECIFIED
1580	MALIGNANT NEOPLASM OF RETROPERITONEUM
1589	MALIGNANT NEOPLASM OF PERITONEUM, UNSPECIFIED
1629	MALIGNANT NEOPLASM OF BRONCHUS AND LUNG, UNSPECIFIED
1642	MALIGNANT NEOPLASM OF ANTERIOR MEDIASTINUM
1649	MALIGNANT NEOPLASM OF MEDIASTINUM, PART UNSPECIFIED
1701	MALIGNANT NEOPLASM OF MANDIBLE

1702	MAL.NEOPLASM OF VERTEBRAL COLUMN, EXCLUDING SACRUM & COCCYX
1703	MALIGNANT NEOPLASM OF RIBS, STERNUM, AND CLAVICLE
1704	MALIGNANT NEOPLASM OF SCAPULA AND LONG BONES OF UPPER LIMB
1706	MALIGNANT NEOPLASM OF PELVIC BONES, SACRUM, AND COCCYX
1707	MALIGNANT NEOPLASM OF LONG BONES OF LOWER LIMB
1708	MALIGNANT NEOPLASM OF SHORT BONES OF LOWER LIMB
1709	MAL.NEOPLASM OF BONE AND ARTICULAR CARTILAGE, SITE UNSPEC.
1710	MAL.NEOPLASM OF CONNECTIVE AND SOFT TISSUE,HEAD,FACE,NECK
1712	MAL.NEOPLASM CONNEC.AND SOFT TISSUE,UPPER LIMB,INCL.SHOULDER
2462	CYST OF THYROID
27788	TUMOR LYSIS SYNDROME
28522	ANEMIA IN NEOPLASTIC DISEASE
